# Clinical, Genetics, and Bioinformatic Characterization of Mutations Affecting an Essential Region of *PLS3* in Patients with BMND18

**DOI:** 10.1155/2018/8953217

**Published:** 2018-10-14

**Authors:** Ting Chen, Haiying Wu, Chenxi Zhang, Jiarong Feng, Linqi Chen, Rongrong Xie, Fengyun Wang, Xiuli Chen, Huiting Zhou, Hui Sun, Fei Xiao

**Affiliations:** ^1^Department of Endocrinology, Metabolism, and Genetic Diseases, Children's Hospital of Soochow University, Suzhou, Jiangsu, China; ^2^School of Biology & Basic Medical Sciences, Medical College of Soochow University, Soochow University, Suzhou, Jiangsu, China; ^3^Institute of Pediatric Research, Children's Hospital of Soochow University, Suzhou, Jiangsu, China

## Abstract

**Background:**

Bone mineral density quantitative trait locus 18 (BMND18, OMIM #300910) is a type of early-onset osteogenesis imperfecta (OI) caused by loss-of-function mutations in the PLS3 gene, which encodes plastin-3, a key protein in the formation of actin bundles throughout the cytoskeleton. Here, we report a patient with PLS3 mutation caused BMND18 and evaluated all the reported disease-causing mutations by bioinformatic analysis.

**Methods:**

Targeted gene sequencing was performed to find the disease-causing mutation in our patient. Bioinformatic analyses mainly including homology modelling and molecular dynamics stimulation were conducted to explore the impact of the previously reported mutations on plastin-3.

**Results:**

Gene sequencing showed a novel nonsense mutation (c.745G > T, p.E249X), which locates at a highly conserved region containing residues p.240–266 (LOOP-1) in the PLS3 gene. Further bioinformatic analyses of the previously reported mutations revealed that LOOP-1 is predicted to physically connect the calponin-homology 1 (CH1) and CH2 domains of the ABD1 fragment and spatially locates within the interface of ABD1 and ABD2. It is crucial to the conformation transition and actin-binding function of plastin-3.

**Conclusions:**

This report identified a novel mutation that truncates the PLS3 gene. Moreover, bioinformatic analyses of the previous reported mutations in *PLS3* gene lead us to find a critical LOOP-1 region of plastin-3 mutations at which may be detrimental to the integral conformation of plastin-3 and thus affect its binding to actin filament.

## 1. Introduction

Bone mineral density quantitative trait locus 18 (BMND18, OMIM #300910), a recently described form of X-linked osteogenesis imperfecta (OI) [1], is caused by loss-of-function mutations of the *PLS3* gene. Male family members with the hemizygous mutation in the *PLS3* gene usually present osteoporosis and fractures of the axial and appendicular skeleton in childhood, while female family members with heterozygous mutations have a wide variety of presentations, ranging from normal bone density and an absence of fractures to early-onset osteoporosis and vertebral compression fractures [2–6].

*PLS3* gene encodes plastin-3, which is a key protein in the formation of actin bundles throughout the cytoskeleton. Its structure is highly conserved from yeast to human and consists of two N-terminal EF-hand Ca^2+^-binding domains and two tandem repeats of C-terminal ABDs (ABD1 and ABD2). Each ABD comprises of a pair of calponin-homology (CH) domains [7–9]. In mammals, plastin-3 is expressed in a cell-type-specific manner and generally manifests in solid tissue, such as neurons, auditory hair cells, melanocytes, and osteoblasts [8]. Defects of plastin-3 in the organization of actin filament bundles in bone cells may interfere the necessary conversion of mechanical signals to biochemical signals in osteoblasts and osteoclasts, thereby leading to osteoporosis [9]. On the other hand, in spinal muscular atrophy (SMA), *PLS3* gene is an age- and sex-specific modifier. PLS3 is important for axonogenesis, and overexpression of PLS3 levels may elevate F-actin amounts and rescue the axonal length and outgrowth defects in SMA patients [10–12].

Almost all reported pathogenic mutations of *PLS3* are inherited from one parent of a patient. They have been observed to be randomly distributed within the gene, and no “hot spot” regions have been found. To date, 11 mutations of *PLS3* have already been identified, distributing over exon 3, 7, 8, 10, 13, 15, and intron 2 [2–6] (see Table S1 in Supplementary Materials). Among these mutations, seven can lead to premature termination codons, two are point mutations, and two are small insertions.

In the present study, we described the first Chinese boy with BMND18 and a novel pathogenic nonsense mutation of the *PLS3* gene [13]. This mutation locates at a highly conserved region containing residues p.240–266 (LOOP-1) in the PLS3 gene. To fully understand the importance of LOOP-1, we studied all previously reported mutations of the *PLS3* gene. By homology modelling, we constructed models of WT and mutant plastin-3 and found that the LOOP-1 region connects the CH1 and CH2 domains of the ABD1 fragment and spatially locates within the ABD1 and ABD2 interface. Further bioinformatic analyses of previously reported mutations of *PLS3* gene revealed that LOOP-1 region was sensitive to conformation transition. Any mutations in this critical region may interfere the integral conformation of plastin-3 and thereby interrupting its conformation rearrangement and binding to actin filament.

## 2. Material and Methods

### 2.1. Subjects

**Figure 1:**
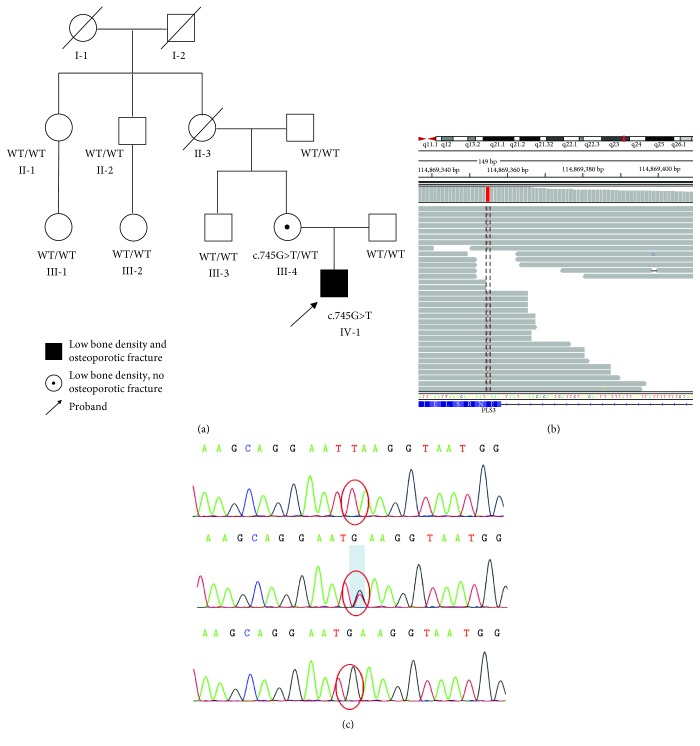
Genetic characterization of the patient described. (a) Family pedigree. Mutation status of *PLS3* c.745G > T is indicated beneath symbols for each subject. WT indicates wild-type. (b) Schematic representation of the mapped exome sequencing reads visualized using the Integrative Genomics Viewer (IGV) browser for the proband and his parents. The red lines indicate the mutation. (c) Sanger sequencing of three individuals in the family over the c.745 position in *PLS3*. The upper panel shows the c.745G > T variant in the patient, the middle panel shows c.745G > T mutation in the heterozygous mother, and the lower panel shows WT in the patient's father.

The patient reported in this study enrolled in the Endocrinology and Metabolism Department of Children's Hospital of Soochow University (Suzhou, China). Informed consent was obtained from the patient, and his family members were included in our study, following procedures specified by the Ethics Committee of The Children's Hospital of Soochow University. The boy, his mother, and his uncle (III-3, see Figure 1(a)) underwent clinical examination, blood sampling, skeletal radiography, and bone densitometry by dual-energy X-ray absorptiometry (DXA). Blood samples for genetic analysis were obtained from the boy and 8 family members (see Figure 1(a)).

### 2.2. Biochemistry

The blood samples were obtained between 7 : 00 a.m. and 8 : 00 a.m. after the subjects had fasted overnight. Serum alkaline phosphatase activity was measured using a standard laboratory method (DiaSys Diagnostic Systems, Germany). Serum 25-OH vitamin D was measured by electro chemiluminescence (Roche Diagnostics, Mannheim, Switzerland). The results were compared to age- and sex-specific reference data.

### 2.3. Bone Densitometry

Dual-energy X-ray absorptiometry was performed in the anterior-posterior direction at the lumbar spine (L1-L4) (GE Healthcare, Madison, Wis). The L1-L4 lumbar spine BMD and femoral neck BMD results were transformed to age- and gender-specific z-scores using reference data. However, as no paediatric reference standards for lumbar spine and femoral neck BMD at this age were available in China, Canadian standards were used [14].

### 2.4. Focused Exome Sequencing

After obtaining informed consent from the boy and his parents, an EDTA-anticoagulated venous blood sample was collected. Genomic DNA was then extracted from peripheral blood lymphocytes by standard procedures using whole-blood DNA extraction kits (BioTeke, Beijing, China). Focused exome sequencing was performed using the target gene panel (SureSelect XT2 focused Exome, Agilent Technologies, Santa Clara, CA, USA) and the HiSeq 2500 sequencing platform. The gene panel contains all reported genes related to OI (COL1A1, COL1A2, IFITM5, SERPINF1, CRTAP, LEPRE1, PPIB, SERPINH1, FKBP10, PLOD2, BMP1, C-propeptide cleavage site, SP7/OXS, WNT1, TMEM38B, CREBL1, and PLS3). BWA (v0.7.7-r441) was used to align the sequence reads to the hg19 reference genome. GATK (v3.1-1-g07a4bf8) was used to call SNV and indels. Ninety-nine percent of the targeted bases were covered at >20x. Finally, the sequence traces were aligned with GenBank reference sequence NM_001136025.

### 2.5. Sanger Sequencing

To confirm the variants found by the exome sequencing of the proband and his family members, Sanger sequencing was performed as previously described. The primer sequences were Fw: 5′-GTGAAGGGAGAAAGTAGACTGCT -3′ and Rev: 5′-TCTCTCTGTGCTTTTGTGTCA-3′. PCR was performed in an ABI 9700 Thermal Cycler using standard conditions. After purification by a QIAquick PCR Purification Kit (Qiagen), the products were directly sequenced onto an Applied Biosystems 3730xl automated sequencer (Life Technologies Corporation Carlsbad, CA, USA). Sequence comparisons and analyses were performed using the Phred-Phrap-Consed program.

### 2.6. Sequence Retrieval, Homology Modelling, and In Silico Mutagenesis

The protein sequence of human plastin-3 was retrieved from the UniProtKB database [15]. However, a suitable template with sufficient query sequence length coverage and sequence identity was unavailable. Therefore, a segment-based homology modelling approach was used in our study. BLAST and PSI BLAST were used for the selection of PDB templates, and templates with more than 40% sequence identity comparing to the target sequence were selected (Table S2). Disordered LOOP-1 was reconstructed in the modelled structures of plastin-3 by applying spatial restraints and energy minimization using MODELLER 9.16 [16]. For each mutant structure, a similar structure preparation procedure was implemented as the wild plastin-3. Subsequently, the target sequence and templates were aligned using MODELLER, and a 3D model was constructed. The constructed model of wild plastin-3 was energy minimized in a GROMACS force field using Steepest Descent Minimization Algorithms followed by a 200 ns molecular dynamics simulation. After geometry optimization, most structural features could be observed in both wild plastin-3 and mutants.

The model of wild type plastin-3 was evaluated by PROCHECK, Root-Mean-Square Deviations (RMSDs), and Root-Mean-Square Fluctuation (RMSF) calculations. PROCHECK validated the model for covalent bond distances, angles, atom nomenclature, and stereo-chemistry [17]. The RMSDs and RMSFs of the C*α* were calculated from the trajectories using the initial structure as a reference. They were good indicators of the uncertainty in the atomic coordinates, and their favourable values were within 0.8 nm. We used PyMOL (version 0.99; DeLano Scientific, San Carlos, CA, USA) for visualizing 3D structures.

### 2.7. Molecular Dynamics Simulations

The predicted structures generated from the homology-based method were used as the starting structures for a molecular dynamics (MD) simulation. All calculations were performed by the GPU-enabled version of the GROMACS4.6.5 package [18] with the AMBER99SB-ILDN force field [19]. The structures were immersed in an octahedral box filled with TIP3P water molecules, imposing a minimum distance between the solute and the box of 15 Å. The charges were then neutralized in electrostatically favourable positions by adding 17 NA+ counter ions to the solvated systems. To eliminate any unfavourable contacts, energy minimization was performed. Each complex was first minimized with the steepest descent algorithm by 5000 steps, and all atom cages were restrained to relax the water molecules and ions. These cages were then minimized with the L-BFGS algorithm without any restraint to relax the entire system. On the minimized structures, a thermalization procedure using the NVT ensemble was conducted by gradually heating the systems from 0 K to 300 K within 30 ps. The optimized systems were then simulated using the NVT ensemble with a 2.0 fs time-step. Periodic boundary conditions were imposed on all studied systems, and 200 ns long trajectories were generated for WT plastin-3 and mutant plastin-3 (c.748ins12AA), respectively, aiming to produce stable protein structure. The electrostatic interactions were calculated every 5.0 fs using a cut-off of 10 Å for the evaluation of short-range nonbonded interactions and the PME method for the long-range electrostatic interactions. The LINCS algorithm [20] was used to constrain the hydrogen-contained bonds. The temperature was fixed at 300 K using V-rescale dynamics [21]. The atomic positions were saved every 5000 steps (10 ps) for analyses.

## 3. Results

### 3.1. Patient

This study reported an 11-year-old male patient with recurrent fractures born to nonconsanguineous parents; both are healthy with negative fracture histories. The boy's prenatal history was not significant. He was born at full-term by vaginal delivery, with a birth weight of 3.4 kg. His cognitive and motor development was normal, and he had a normal gait pattern during childhood. The first fracture (left humerus) occurred at age 6 after a fall. One month before his referral, he suffered another fracture (left distal radius).

Subsequent physical examination of the proband revealed blue sclera. He had a normal height (146.9 cm, about 60^th^ percentile), gait pattern, and muscle tone. His L1-L4 BMD (0.514 g/cm^2^, z-score −1.2) and femoral neck BMD (0.531 g/cm^2^, z-score −2.1) were low, and a spine X-ray showed multiple thoracic vertebral compression fractures (Figure S1). However, as no paediatric reference standards for lumbar spine and femoral neck BMD were available in China at the time of this study, we used Canadian standards that may be inappropriate for our patient [14]. The proband's mother showed low BMD of the femoral neck (0.764 g/cm^3^, z-score −1.4, T-score −1.6) and no spinal compression fractures (Table 1).

### 3.2. Identification of the P. Glu245^∗^ Mutation

Focused exome sequencing of the proband revealed a novel nonsense mutation in exon 7 of the *PLS3* gene (c.745G > T). According to ACMG standards and guidelines, the mutation E249X has one very strong evidence (nonsense mutation), one strong evidence (De novo mutation), and one moderate evidence (absent in 1000G and ESP6500 database). Therefore, this mutation is pathogenic [13]. No mutations were found in other OI-related genes. Sanger sequencing confirmed that while the proband is hemizygous for this mutation, his mother is heterozygous for this mutation. All other family members tested do not carry this mutation (Figure 1). This mutation (E249X) will cause nonsense-mediated decay of mRNA. As a result, theoretically, no PLS3 protein will be produced in the patient.

### 3.3. Sequence Conservation Analysis

**Figure 2:**
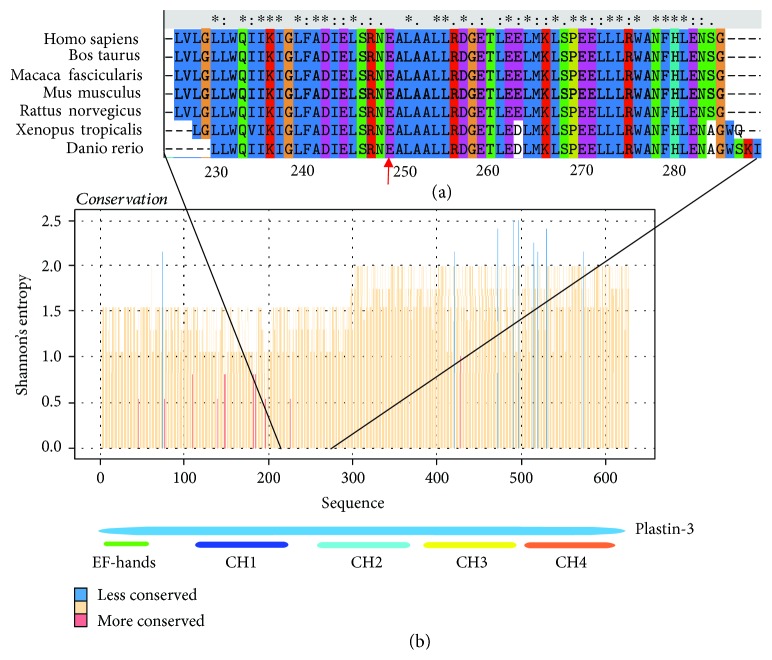
Sequence conservation in the plastin-3 family. (a) Local view of the multiple sequence alignment of plastin-3 in the neighbourhood of Glu249 (red arrow). (b) Whole plastin-3 plot. Conservation is represented using Shannon entropy, a parameter routinely used for scoring pathogenic mutations, whose values range from 0 (complete conservation) to 4.32 (all amino acids are equiprobable at that position).

Sequencing of the *PLS3* gene showed a region containing the residues p.240–266 with a high degree of conservation, which we named LOOP-1. This region physically connected to the CH1 and CH2 domains and was spatially located at the ABD1-ABD2 interface. This finding suggests that the LOOP-1 region is a universally and biologically relevant feature among various species (Figure 2). Including our case, three reported mutations were located at LOOP-1 (Table 1). Among them, two (p. Ala253_Leu254insAsn and p. Glu249_Ala250ins12) may cause small insertions within LOOP-1.

### 3.4. Structure Modelling and Analysis

**Figure 3:**
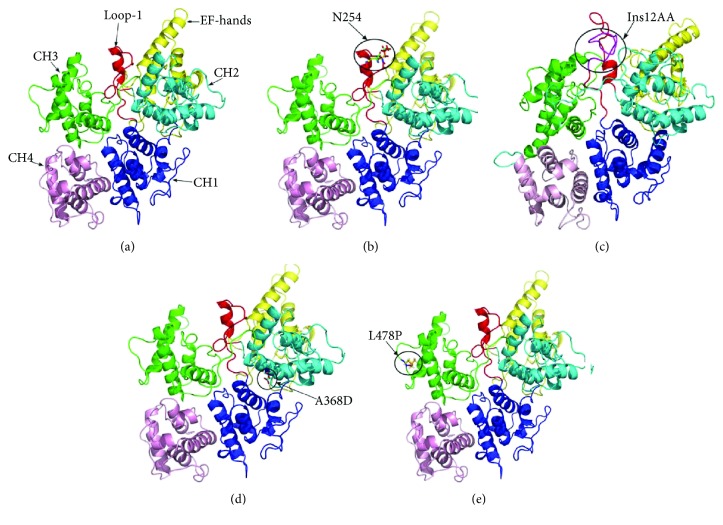
Structures of wild-type and mutant plastin-3. The run of the protein chain is shown as a secondary structure cartoon color-coded according to five domains. (a) Classical view of the structure of the wild-type plastin-3. The EF-hand domains are shown in yellow, CH1 in blue, CH2 in cyan, CH3 in green, CH4 in pink, and the LOOP-1 region in red, respectively. Mutant plastin-3 structures are shown in (b) (c.759_760 ins AAT), (c) (c.748ins36), (d) (c.1103C > A), and (e) (c.1433G > A), respectively. The location of the mutated residues is highlighted in black circle, and residues are highlighted in sticks.

To date, 11 mutations of the *PLS3* gene have been discovered in BMND18 patients, 7 of which led to premature termination codon. To understand the molecular repercussions of the mutations related to plastin-3 function, this study reconstructed the WT and 4 mutant structures (2 missense mutations and 2 small insertion mutations) of plastin-3 through homology modelling. As shown in Figure 3(a), the structure of WT plastin-3 consists of two N-terminal EF-hand Ca^2+^-binding domains and two tandem repeats of two C-terminal ABDs (ABD1 and ABD2). Each ABD is composed of a pair of calponin-homology (CH) domains [7, 9]. Two insertion mutations were found at the LOOP-1 region (Figures 3(b) and 3(c)), which was located within the CH2 and CH3 interface. The mutation c.1103C > A (p. A368D) was buried at the interface between CH1 and CH2 (Figure 3(d)), and the mutation c.1433G > A (p. L478P) in the CH3 domain was located at the actin-binding interface as previously described (Figure 3(e)) [22].

### 3.5. Dynamics of WT and Mutant (Glu249_Ala250ins12) Plastin-3

The mutation Glu249_Ala250ins12 located at the LOOP-1 region was shown to significantly fluctuate the structures of plastin-3. Accordingly, the mutant structure of Glu249_Ala250ins12 was taken as an example to investigate how mutations within the LOOP-1 region affect the dynamics of the structures.

**Figure 4:**
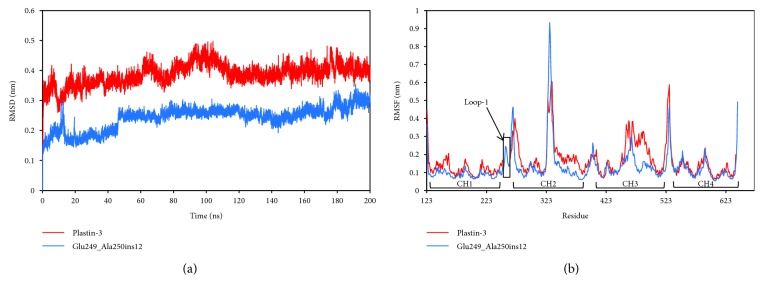
The RMSD is calculated on C*α* atoms of the wild-type plastin-3 (red) and the mutant (Glu249_Ala250ins12) (blue) structure (a), respectively. (b) Atomic fluctuations (RMSF) is calculated from the C*α* of the wild-type (red) and mutant (blue) plastin-3 during MD simulations.

To assess the stability of MD trajectories of both WT and mutant structures, the RMSDs of the C*α* coordinates as a function of a sequential time series (taken every 10 ps) were calculated with respect to the first trajectory frame, as shown in Figure 4. The RMSD values of WT plastin-3 showed a greater deviation (0.39 ± 0.04 nm) than that of the mutant system (0.24 ± 0.04 nm) (Figure 4(a)), indicating that this model undergoes larger conformation change relative to the initial structure. The RMSF values show a similar trend as the RMSD results (Figure 4(b)), which shows that WT plastin-3 undergoes a more obvious conformation fluctuation than the mutant system, especially for the CH2 and CH3 domains.

**Figure 5:**
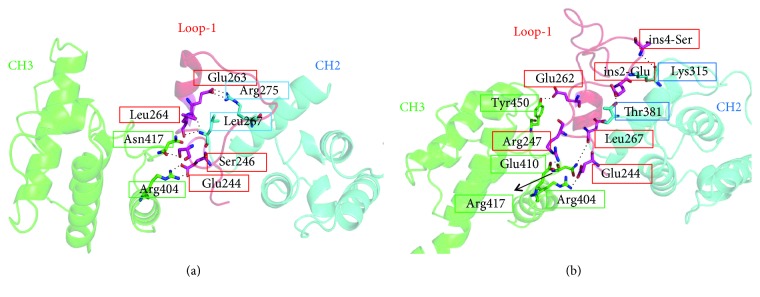
The hydrogen bonds formed between the CH2 and CH3 interface for wild plastin-3 system (a) and Glu249_Ala250ins12 system (b), respectively. The protein is represented with colored cartoon, red for the LOOP-1 region, blue for CH2 domain, and green for CH3 domain, respectively. Residues involved in the h-bonds interactions are highlighted with sticks.

To understand the atomic level influence of Glu249_Ala250ins12 on the CH2 and CH3 interface, the hydrogen bonds (h-bonds) formed between residues of LOOP-1 and CH2/CH3 domains were analyzed during the MD simulation (see Figure 5 and Table S3). There were significant differences in the pattern and strength of h-bonds between the WT and mutant system. Interestingly, we discovered that the h-bonds between LOOP-1 and CH2 domain were obviously weakened, while the h-bonds between LOOP-1 and CH3 domain were significantly strengthened in the mutant system. Therefore, insertion of 12 amino acids between Glu249 and Ala250 within LOOP-1 region strengthened the compactness of the CH2 and CH3 interface, thereby making ABD1 and ABD2 a more compact conformation than those of WT plastin-3.

## 4. Discussion

This study was first to describe a Chinese boy with BMND18. Focused exome sequencing of the boy revealed a novel nonsense mutation (c.745G > T) in the *PLS3* gene that has not been reported previously. Sanger sequencing confirmed that the boy was hemizygous for this mutation, and his mother, who has osteopenia but no osteoporotic fractures, was heterozygous for this mutation. The mutation c. 745G > T causes a premature stop codon in a highly conserved region of ABD1, then results in the truncation of plastin-3. This mutation cannot be found in any population database. Therefore, according to the ACMG guidelines, this mutation is pathogenic [13].

The phenotypes of our patient were consistent with previously reported BMND18 cases [2–6]. No extraskeletal manifestations were observed in our patient, except blue sclera. This phenotype is a rare phenotype in BMND18 patients but has also been observed before [5]. In 2016, Nishi et al. reported two brothers with a missense mutation (c.1103C > A) of the *PLS3* gene presenting developmental delay, deafness, low mineral density, and blue sclera. Therefore, blue sclera may be a minor phenotype of BMND18 and cannot be used to distinguish BMND18 from other types of OI.

Plastins are actin-binding proteins that crosslink filamentous actin into compact ordered bundles presenting in different cytoskeletal processes [23]. The mechanism that describes how ABDs of plastin-3 interact with actin remains unclear. The mutation c.745G > T we found in the boy with BMND18 locates at a highly conserved region containing residues p.240–266 (LOOP-1), which we think may of great importance to the function of PLS3. To fully understand the importance of LOOP-1, we studied all previously reported mutations of the *PLS3* gene via bioinformatic analysis. Besides seven mutations that can cause premature termination codon, we found two point mutations and two small insertions. These two small insertions were both located at LOOP-1, which physically connects CH1 and CH2 domains together and are spatially located at the interface between ABD1 and ABD2. As shown in Figure 2, the LOOP-1 region is highly conserved among species. Besides, as shown in Figure S2, the connection region between the CH1 and CH2 domains is unorganized, thereby indicating that these segments are highly dynamic.

According to the previously reported cryo-EM model of plastin-3, the structural conservation of the CH-domains was not observed to lead to a conserved mode of ABDs interaction with F-actin [22]. The binding of ABD1 to F-actin is partial and disordered, while the binding of ABD2 to F-actin is stoichiometric and ordered. It has been proposed that ABD2 can bind first to one F-actin filament, which in turn “activates”ABD1 and enables it to bind in a more ordered manner to another actin filament with a higher affinity [21]. This cooperation between the two ABDs is conducted by the interface between ABD1 and ABD2 [24]. Therefore, we suppose that the LOOP-1 region located at the interface between ABD1 and ABD2 may be structurally and functionally critical in the cooperation between two ABDs.

To investigate this idea, the mutant structure of Glu249_Ala250ins12 was constructed to compare with WT plastin-3. Molecular dynamics stimulation and hydrogen bond analysis both suggested that insertion of 12 amino acids in the LOOP-1 region can increase the compactness between CH2 and CH3 domains, thereby interfering the conformation rearrangement between ABD1 and ABD2. This result supports the hypothesis that the LOOP-1 region has a functionally relevant role, resulting from its contribution to the “active” state of ABDs. Mutations at this location may have a disruptive effect on the ABD interface, thereby affecting the activity of ABDs binding to F-actin [7, 24, 25].

In summary, this study presented the first case of BMND18 with a novel *PLS3* gene mutation in a Chinese male patient. The in silico studies of the present and previous cases of BMND18 helped us to identify a critical LOOP-1 region. Mutations within LOOP-1 may change the compact conformation and activation of ABDs and thus disrupt the function of plastin-3. Further experimental studies are required to explore the function of LOOP-1 in the binding of plastin-3 to F-actin.

## Figures and Tables

**Table 1:** Clinical characteristics, BMD scores, and biochemistry at the time of the first evaluation.

Subjects	IV-1	III-4	III-3
	Hemizygous	Heterozygous	No mutation
Age	11	40	44
Sex	Male	Female	Male
Long-bone fractures, *n*	2	0	0
Vertebral compression fractures	Yes	No	No
L1-L4 BMD (z-score)	−1.2	0	0.8
Femoral neck BMD (z-score)	−2.1	−1.4	0.5
Alkaline phosphatase (IU/L)	252	49	36
25-OH vitamin D (nmol/L)	23	31	45

## Data Availability

The data used to support the findings of this study are included within the article.

## Supplementary Materials

Supplementary Materials We have two supplementary files. The supplementary word file contains three supplementary tables and two supplementary tables described in the text that support our results. Table S1: list of all the mutations on gene PLS3. Table S2: list of PDB templates used for segment-based homology modelling. Table S3: comparison of the hydrogen bonds formed by residues of LOOP-1 and CH2/CH3 domains in the wild-type and mutant (Glu249_Ala250ins12) plastin-3, respectively. Figure S1: the spine radiograph of the proband with the PLS3 (c.745 G > T) mutation. Figure S2: the ribbon rendering of the carbon backbone of the PLS3 core model (ABD1 and ABD2 are cyan and green, respectively) is shown within a semitransparent representation of the molecular surface. The supplementary excel file contains all the variants detected in the patient via focused exome sequencing.
