# Supplementary material for: Clinical, Genetics, and Bioinformatic Characterization of Mutations Affecting an Essential Region of PLS3 in Patients with BMND18
Source: Int J Endocrinol. 2018 Oct 14;2018:8953217. doi: 10.1155/2018/8953217 (PMC6204236; doi:10.1155/2018/8953217)
Supplement: Supplementary Materials — We have two supplementary files. The supplementary word file contains three supplementary tables and two supplementary tables described in the text that support our results. Table S1: list of all the mutations on gene PLS3. Table S2: list of PDB templates used for segment-based homology modelling. Table S3: comparison of the hydrogen bonds formed by residues of LOOP-1 and CH2/CH3 domains in the wild-type and mutant (Glu249_Ala250ins12) plastin-3, respectively. Figure S1: the spine radiograph of the proband with the PLS3 (c.745 G > T) mutation. Figure S2: the ribbon rendering of the carbon backbone of the PLS3 core model (ABD1 and ABD2 are cyan and green, respectively) is shown within a semitransparent representation of the molecular surface. The supplementary excel file contains all the variants detected in the patient via focused exome sequencing. [file 8953217.f1.zip › mat.8953217.v2 (1).docx]

Supplementary Materials

Table S1. List of all the mutations on gene PLS3^2-6^

| Catagory | Nucleotide change | Amino Acid change |
| --- | --- | --- |
| Missense mutation | c.1103 C>A | p.Ala368Asp |
|  | c.1433 T>C | p.Leu478Pro |
| Nonsense mutation | c.1471 C>T | p.Gln491* |
|  | c.745G>T | p.Glu249* |
| Small deletion | c.235del T | p.Tyr79Ilefs*6 |
|  | c.994_995del GA | p.Asp332* |
|  | c.1730dup T | p.Leu577* |
|  | c.1647delC | p.Ser550Alafs*9 |
| Insertion | c.759_760insAAT | p.Ala253_Leu254insAsn |
| Splice site mutation | c.73-24 T>A | p.Asp25Alafs*17 |
|  | c.758+1 G>A | p.Glu249_Ala250ins12 |
| Microdeletion | Del p23(112,419,139–  115,830,286):3.411MB | – |

Table S2. List of PDB templates used for segment based homology modelling.

| **Segment based Homology Modeling** | | | |
| --- | --- | --- | --- |
| **Tools used/purpose** | **Selected templates (PDB ID)** | **Length covered by PDB template on the protein sequence** | **Sequence/ fold identity between the PDB**  **and reference protein sequence** |
| BLAST & PSI BLAST  (Selection of PDB templates for model construction) | 1AOA | 101-375 | 100% |
|  | 1RT8_A | 119-624 | 44% |
|  | 1PXY_A | 123-629 | 42% |
|  | 1WJO_A | 516-630 | 97% |
|  | 2D85_A | 516-630 | 78% |
|  | 5JOL_A | 5-85 | 56% |
|  | 5JOJ_A | 5-100 | 59% |

Table S3. Comparison of the hydrogen bonds formed by residues of LOOP-1 and CH2/CH3 domains, in the wildtype and mutant (Glu249_Ala250ins12) plastin-3, respectively. Percentage >20% has been considered.

| **Location** | | | **plastin 3** | | | | **Glu249_Ala250ins12** | |
| --- | --- | --- | --- | --- | --- | --- | --- | --- |
|  |  |  | **Hydrogen bond contact** | | | **percentage** | **Hydrogen bond contact** | **Percentage** |
| **LOOP-1**  **and**  **CH2** | | | Asp242 Asn385 | | | 0.54 | ins4-His Gln320 | 0.28 |
|  |  |  | Arg247 Asp389 | | | 0.55 | Ins5-Ser Lys482 | 0.24 |
|  |  |  | Arg247 Glu384 | | | 0.43 | Glu275 Arg287 | 0.21 |
|  |  |  | Glu249 Thr381 | | | 0.22 |  |  |
|  |  |  | Glu259 Arg275 | | | 0.96 |  |  |
| **Tota**l | | | |  | | 2.70 |  | 0.73 |
|  |  | | | |  |  | Asp242 Arg413 | 0.30 |
| **LOOP-1** | | | | Glu244 Arg404 | | 1.49 | Glu244 Arg413 | 0.20 |
| **and** | | | |  | |  | Glu244 Arg416 | 1.54 |
| **CH3** | | | |  | |  | Arg247 Glu412 | 1.52 |
| **Total** | |  | | | | 1.49 |  | 3.56 |


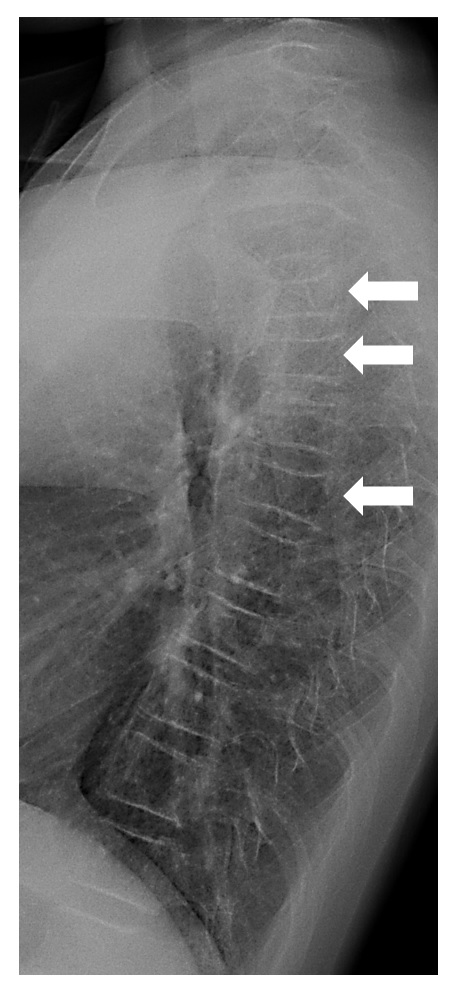


Figure S1. The spine radiograph of the proband with the *PLS3* (c.745 G>T) mutation. He had vertebral compression fractures and osteoporotic appearance of the vertebrae.


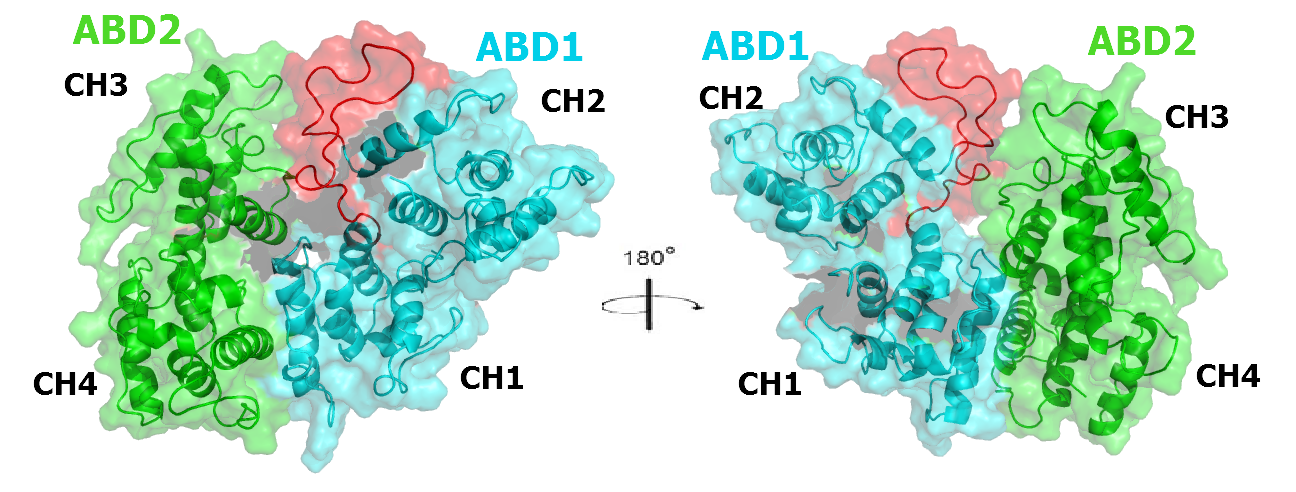


Figure S2. The ribbon rendering of the carbon backbone of the PLS3 core model (ABD1 and ABD2 are cyan and green, respectively) is shown within a semitransparent representation of the molecular surface. The residues corresponding to the connection between ABD1 and ABD2 domains are mapped onto the core structure and colored red. These residues are buried in the CH2/CH3 interface, and several of them are inaccessible to solvent. B. Semitransparent representation of the molecular surface with ribbon rendering of the carbon backbone of the PLS3 rotated 180º about a vertical axis relative to A. This representation highlights the different surfaces of both ABDs presenting to solvent.
